# Protocol Independent Adaptive Route Update for VANET

**DOI:** 10.1155/2014/403918

**Published:** 2014-02-27

**Authors:** Asim Rasheed, Sana Ajmal, Amir Qayyum

**Affiliations:** ^1^Muhammad Ali Jinnah University, Islamabad, Pakistan; ^2^Center for Advanced Studies in Engineering, Islamabad, Pakistan

## Abstract

High relative node velocity and high active node density have presented challenges to existing routing approaches within highly scaled ad hoc wireless networks, such as Vehicular Ad hoc Networks (VANET). Efficient routing requires finding optimum route with minimum delay, updating it on availability of a better one, and repairing it on link breakages. Current routing protocols are generally focused on finding and maintaining an efficient route, with very less emphasis on route update. Adaptive route update usually becomes impractical for dense networks due to large routing overheads. This paper presents an adaptive route update approach which can provide solution for any baseline routing protocol. The proposed adaptation eliminates the classification of *reactive* and *proactive* by categorizing them as *logical conditions* to find and update the route.

## 1. Introduction

Dynamics of wireless networks have changed due to round the clock data connectivity requirements, all over the place. New and specialized data networks have emerged, requiring high mobility and scalability. These networks may have disconnected topologies, sudden change in active node densities, and broadcast storms. Specialized and complex deployment and movement patterns in wide areas challenge QoS support. Vehicular Ad hoc Networks (VANETs) are a case of the highly fluent wireless networks [1, 5, 14, 22].

Efficient routing aims finding optimum route, updating it on availability of better one, and then maintaining it, by keeping low overheads. Accordingly, researchers have proposed a number of routing protocols using a variety of metrics, for example, hop count, node location, and so forth [2, 6, 20, 23, 24, 28, 32]. Accordingly, route finding and maintenance are done through the following approaches:

1. periodic metrics sharing (proactive routing),
2. event based metrics sharing (reactive routing),
3. derivatives of (1) and (2), for example, hybrid or history oriented approach.

Route update on availability of a better one requires updated information of network conditions. Considering the definition, route update is technically not possible for event triggered routing. In such protocols, route is only updated once the old route fails or a new connection or packet exchange is initiated. At the same time, the periodic update approach may add significant overheads to the network traffic. The approach (proactive or reactive) is generally fixed and predefined in the protocol instead of being based on runtime network conditions. Adaptation of routes has been proposed by researchers through multiple approaches, for example [20, 28, 32]:

- the inclusion or exclusion of a specific node from the route according to the changes in runtime conditions, such as traffic load, mobility, or node density. This approach is generally used for energy conservation or load balancing [32],
- switching between precomputed multiple routes according to runtime conditions, known as Adaptive Multipath Routing [7],
- proactive updating of routes using geographical locations and so forth.

For Adaptive Multipath Approach, one observation suggests that as all routes are precomputed, though the best amongst the pool, the selected route may not be holistically the optimum one under given conditions. For proactive updating using geographical locations, the requirement of timely and accurate sharing of updated node locations might incur additional overheads.

Route update strategies must support realistic but diverse deployment, mobility patterns, and QoS requirements. We propose an adaptive route update scheme, independent of the baseline routing algorithm, which uses logical conditions to find and update the route.

The rest of this paper is organized as follows.  Section 2 analyses the current routing strategies.  Section 3 explains the model for adaptive route update.  Section 4 deals with the simulation results and their analysis.  Section 5 concludes the paper.

## 2. Analysis of Current Routing Strategies

Mobile wireless networks suffer from sudden link breakages due to topological changes, changes in node densities, and reduction in end-to-end link capacities. Resultantly, each VANET node requires flexible, generic, and adaptive route update and maintenance strategy [29]. Different factors directly affect the routing strategies, even before route determination mechanism, such as deployment and mobility patterns.  Table 1 explains the effect of different factors on route update strategy. It can be observed that no single routing approach satisfies all conditions. A single node may face a variety of conditions during the same session, such as change in node density and variation in relative node velocity.

In subsequent sections, we have evaluated current routing metric sharing strategies with emphasis on state of the art in VANETs.

### 2.1. Simple Evaluation of Current Metric Sharing Approaches

To observe the effect of increased routing overheads for proactive routing protocols, state-of-the-art OLSRv2 [34] protocol was tested for high node densities. We computed throughput using simple but realistic node topologies with high throughput MAC protocol IEEE 802.11n. To start with, two mobile nodes from two hop distance were moved towards each other. While staying in direct communication, node density was gradually increased for more medium contention.

On increasing the node density, significant increase in routing as well as total overheads was observed at each node. With the increase in the number of nodes, number of routing messages as well as size of HELLO packets was also increased. Due to increased control as well as data packets, each node faced medium congestion. The increase in transmission retries also added overheads. After increasing the node density to 86 nodes, we observed that total overheads approached the actual application throughput for each node. For such high rate of overheads, randomly selected nodes faced lack of communication resources for even routing and control packets.

**Figure 1:**
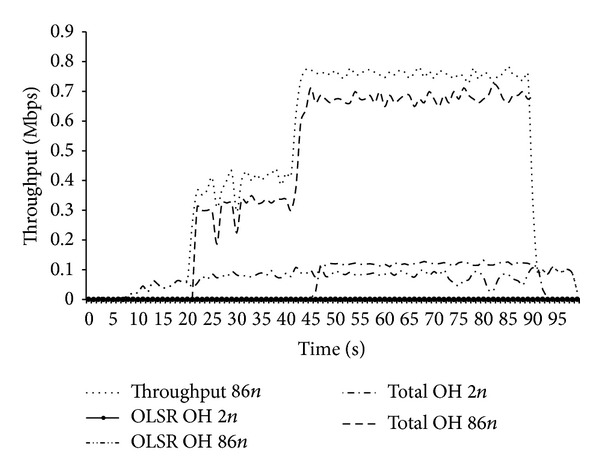
Comparison of per node routing overheads, total overheads, and net throughput.

Figure 1 shows the increase of total overheads with increase in routing overheads. The *x*-axis shows time scale in seconds, whereas *y*-axis shows the per node throughput and overheads in Mbps. Five different curves show comparison between routing overheads, total overheads, and end-to-end throughput among end nodes for 2 and 86 nodes.

The curves in the Figure 1 show that increase in node density also significantly increased routing as well as total overheads. Both overheads increased approximately eight times from initial values. Due to high rate of overheads, many nodes even fail to share the control information. Owing to design limitations, the phenomenon of increased total overhead will replicate for all protocols with proactive metric sharing scheme, regardless of selected metric, that is, node location or link history.

### 2.2. VANET Routing Protocols

With the emergence of new and more complex requirements on routing, new routing schemes are being proposed by researchers at a fast rate. Using a variety of metrics and three basic metric sharing schemes, we have classified the existing routing protocols into the following major categories:

- link state and distance vector MANET routing protocols, such as OLSRv2 and AODVv2 (DYMO), generally perform topology based routing. In many cases, these protocols face performance degradation issues with increased scalability and rapid link breakages [30].
- Broadcast based routing protocols typically flood the data in entire network. Although this approach ensures delivery, it can only work for small scale networks. Modifications of this approach such as V-TRADE and HV-TRADE routing protocols [3] limit the flooding and show improvement over traditional approach by reorganizing the network in subgroups. However there are significant routing overheads for rebroadcasting.
- In overlay routing, the routing protocol operates on a set of representative nodes laid over network topology, for example, GPCR [31] and CAR [4]. In the dense environments (e.g., urban scenarios), street junctions can be used as decision points for subsequent selection of route. Similarly, use of geographical features can also help in decision making for routing in highway scenarios. Appropriate selection of overlay map, for example, junction points, can assist in timely delivery of data using shortest path.
- Cluster based routing, for example, CBRP [15] and COIN [8], is the combination of the above two techniques. In such schemes, each node designates a cluster-head within a subset of nodes. The cluster-head node broadcasts the required packet to cluster members. Although these protocols answer the scalability issue, additional delays and overhead are incurred while forming and maintaining clusters.
- Infrastructure or road side unit (RSU) based routing protocols, for example, RAR [9] and MOVE [25], forms the concept of hybrid networks. Being static in nature, each RSU maintains information about other RSUs and directly connected mobile nodes. Hence, in such networks, maximum reliance is given to RSU for selecting a route to destination.
- Location based routing protocols, for example, CMGR [23] and GPSR [12], are generally claimed to be suitable for highly scaled networks, such as VANETs. The information of nodes all along the path reduces delay in route determination. Use of location information instead of hierarchical routing tables significantly reduces routing overheads. These protocols answer scalability and delay in route determination issues. However, lack of updated and exact location of all the nodes can degrade the routing performance [10, 21]. They can also suffer from routing loops and disconnected network topologies [12].
- Geocast routing, for example, BBR [17], is a combination of broadcast routing and position based routing. In this scheme, data is broadcast within a specific geographical region around the source. This scheme is useful for control and safety information dissemination. Some other scheme can be used for data transmission outside geographical region. The network partitioning and mapping of geographical regions on road layout are major limitations of this approach.
- Delay tolerant routing, for example, VADD [16] and GeOpps [18], is generally a new concept for the nodes spread in the sparse areas. As establishing an end-to-end route may not be possible in the absence of next hop neighbour under disconnected topologies, packets are buffered till availability of next hop neighbour. This approach is generally known as carry-and-forward.
- A quality of service (QoS) based routing, for example, PBR [33], generally performs resource reservation prior to the start of data transfer. Such guarantees are difficult for highly dynamic networks in a deterministic manner but may be given in a probabilistic sense.

Above stated simple evaluation confirms that current routing approaches with predefined and fixed route update scheme cannot perform, even for the simple high node density scenarios. Such network scenarios are very common for complex networks such as sports stadium and traffic jams. These circumstances can cause absolute routing failure for many successful routing protocols. For these routine situations, node density may even increase to thousands of nodes, highlighting need for optimised and adaptive route update.

## 3. Adaptive Route Update Strategy Model

Researchers have observed that localised route maintenance performs better than end-to-end route repair due to involvement of less number of nodes in the process [2]. For efficiency, all nodes are required to perform local route update on two independent conditions, that is:

- when the link with next hop neighbour is about to break,
- when 2nd hop neighbour comes in direct range.

For the overall analysis, study of the combined impact of both conditions is necessary. The possibility of any neighbour node to affect the host node depends upon two factors:

- in which direction Δ*θ* the neighbour node is moving, relative to host node
- how much distance Δ*d* a neighbour node can cover relative to the host node during time *t*.

Accordingly, Δ*d* can be computed as:

(1) $$\begin{array}{c} \Delta d=\left(\Delta v\right)t=\left(v_{n}\cos\left(\theta_{n}\right)-v_{h}\right)t, \end{array}$$

where *v*_*n*_ is velocity of next hop neighbour, *θ*_*n*_ is the relative angle between the node and its neighbour, *v*_*h*_ is velocity of the host node, and *t* is the lapsed time since last measurement.

The change in topology around any host node *H* can be determined by the movement of two hops neighbouring nodes, during time *t*. Whereas, only nodes capable of covering Δ*d* distance from the maximum hop distance during time *t* will be able to either leave the next hop region or enter in it.

**Figure 2:**
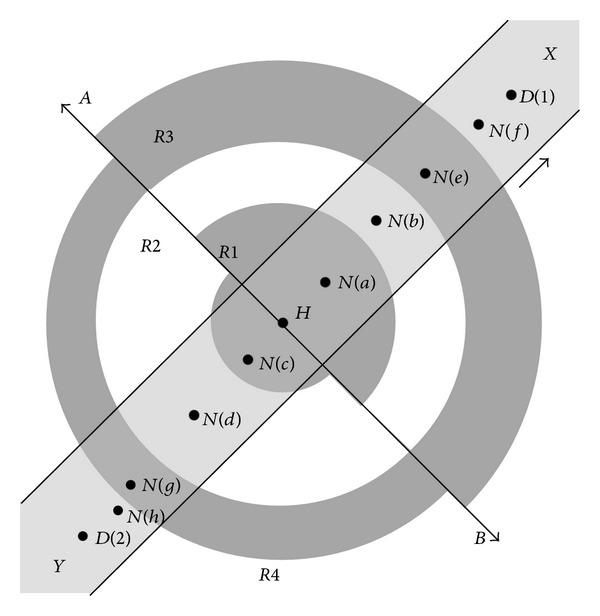
Link ranges for the test topology.

Behaviour of the neighbours can be explained by studying the area around *H*. Using (1), Figure 2 defines different communication ranges with respect to the host node *H*. For a relatively simpler model, we assumed that *H* is moving on strip segment *XY*, for example, nodes moving on a road in VANETs. Segment *XY* is divided into lanes. Direction of arrow shows that the movement direction of *H*. *N*(*a*, *b*, *c*, and *d*) depicts the four next hop neighbours, whereas *N*(*e*, *f*, *g*, and *h*) depicts the four 2nd hop neighbours, moving in the same or opposite direction of node *H*, respectively. *D*(1) and *D*(2) depict the destination nodes.

The probability of selection of next or 2nd hop neighbour, in any region, will vary according to the selected routing metric. The probability of selecting a next hop node closer to the edge, will be more, if the routing metric is minimum hop count. However, the probability will be less for the consideration of maximum stable link. For our model, we can consider different generic probabilities for different regions as:

- *P*(1) is the probability of next hop neighbour in *R*1, for example, nodes *N*(*a*) and *N*(*c*),
- *P*(2) is the probability of next hop neighbour in *R*2, for example, nodes *N*(*b*) and *N*(*d*),
- *P*(3) is the probability of 2nd hop neighbour in *R*3, for example, nodes *N*(*e*) and *N*(*g*),
- *P*(4) is the probability of 2nd hop neighbour in *R*4, for example, nodes *N*(*f*) and *N*(*h*).

For a simple generic model, it can be assumed that mobile nodes within the same lane are moving with almost equal speed. Due to bidirectional behaviour of lanes, nodes can move in line or in opposite direction to *H* or independently adapt static behaviour. Accordingly, variations due to change of speed and direction of movement will create different link stability behaviours. Resultantly, effect of the relative distance Δ*d* and relative angle of movement Δ*θ* between the next and 2nd hop neighbour nodes require detailed analysis.

For better understanding of node movement, Δ*d* and Δ*θ*, we can divide the next hop region into two equal halves, on the axis perpendicular to direction of movement. According to simple geometrical layout of node deployment, we can compare both halves as follows:

- the 2nd hop neighbours, which exist towards the direction of motion of *H*, have a higher probability of coming into direct range of *H*.
- On the other hand next hop neighbour nodes, existing in the half opposite to the direction of motion of *H*, have a high probability of going out of range of *H*.

We assume that the maximum transmission range of the host node is up to the 2nd region (*R*2). Hence, next hop neighbours can exist within first two regions only. Similarly, 2nd hop neighbours can exist outside 2nd region. Moreover, during *t*, two neighbours can cover maximum distance of less than half of communication range of host node. Hence, nodes present in 1st region (*R*1) will remain in communication during *t*.

Due to limited covered distance in time *t*, Nodes within *R*2 will have the chance of link breakage because of its close proximity to the maximum transmission range boundary. In the area opposite to direction of movement of *H* (lower half), neighbour nodes moving in line with *H* but with negative relative velocity will also be able to go out of range from *H*.

Neighbouring nodes within same region, but moving opposite to *H*, will have double dispersion from *H* during time *t*. This dispersion will introduce expansion in *R*2 as compared to upper half.

Similar to *R*2, nodes in the 3rd region (*R*3) will have high probability of coming within the range of *H*. Like *R*2, nodes in *R*3 (upper half) moving opposite to *H* will also have double convergence in *t*. Similar to *R*1, nodes in 4th region (*R*4) will not directly affect *H* in time *t*.

### 3.1. Effect of Δ*d* on Metric Sharing Approaches

Routing protocols with time based metric sharing approach use HELLO packets for assessment of network topology. As defined earlier, only the nodes present in Δ*d* distance from maximum hop boundary can go out of range between two consecutive HELLO intervals (time *t*). Hence sharing of network topology on all nodes during *t* will cause resource wastage for nodes outside Δ*d*. The rate of possible nodes involved in change of topology increases with increase in node speed and duration of HELLO interval.

Reactive metric sharing approach does not perform route update, whereas proactive metric sharing performs route update on all nodes regardless of their involvement in topology change. Hence, these protocols do not perform optimum route update and fail for the complicated scenarios. Although number of adaptive routing protocols are proposed, periodic or event based metric sharing approaches do not consider this important conclusion.

### 3.2. Neighbouring Link Behaviour

For both of the halves depicted in Figure 2, mobility can generate multiple scenarios according to change in relative velocity. According to mobility of next or 2nd hop neighbouring nodes with respect to *H*, four different possible relations are possible as:

- both nodes are static,
- one node is static and other is moving,
- both nodes are moving in same direction,
- both nodes are moving in opposite direction.

To start with, simple relation of two nodes in upper half of Figure 2 can be evaluated.  Table 2 defines the relation between two neighbouring nodes in right half of *R*2 during time *t*. Presence of neighbour node can have six possible scenarios according to our assumptions and mobility relations defined above. Considering the same speed in case of mobility, only one scenario can face link breakage possibility as defined in Table 2. Hence, according to different possible options of mobility and direction of move, link breakage will be possible only for the cases where neighbour node has positive relative mobility.

The same relation can be expanded to multiple lanes for the next hop neighbour inside right half of *R*2. To make a generic relation, we can consider nodes with uniform density.

Considering the different possible scenarios, effect of mobility on link breakage probability can be computed, according to its relation with the number of lanes. Using the results of Table 2, an expression can be formulated for the probability of the next hop neighbour, going out of range of *H*, in time *t*, that is, *P*(*N*_1_(out)), as:

(2) $$\begin{array}{c} P\left(N_{1}\left(\text{out}\right)\right)=\frac{\sum \left(l-a\right)}{l\left(l+1\right)+{\left(l+1\right)}^{2}}, \end{array}$$

where *a* is the dummy variable for the number of lanes *l* and *a* = 0,1, 2,…, (*l* − 1).

From the assumption of uniform node density, we know that number of nodes *n* is directly proportional to the number of lanes *l* and can have the worst case of single node per lane. Hence using (2), the probability of next hop neighbour node existing in upper half of *R*2 and going out of range from host node is:

(3) $$\begin{array}{c} P\left(N_{1}\left(\text{out}\right)\right)=\frac{1}{2}P\left(2\right)\left(\frac{\sum \left(n-a\right)}{n\left(n+1\right)+{\left(n+1\right)}^{2}}\right), \end{array}$$

similarly, in other regions, we can formulate the mathematical expression for probability for next hop neighbour going out of range, that is, *P*(*N*_1_(out)), or for 2nd hop neighbour coming in range, that is, *P*(*N*_2_(in)). Subsequently, the overall probability for optimal route update, conditioned on breakage of the next hop link or establishment of a direct link with the 2nd hop neighbour, can be computed using the combined probability, as:

(4) P[N1(out)  or  N2(in)] =(12n(n+1)+(n+1)2)  ×(((P(2)+P(3))(∑(n−z)+∑(n+y)))    +((P(2)+P(3))n2)−(12n(n+1)+(n+1)2)    ×((P(2)P(3))(∑(n−z)+∑(n+y))+n2)2),

where *y* and *z* are dummy variables for number of nodes, *n*, *z* = 0,1, 2,…, (*n* − 1), and *y* = 0,1, 2,…, *n*. *P*(2) and *P*(3) are the probabilities of any node in *R*2 and *R*3, and *P*[*N*_1_(out)  or  *N*_2_(in)] is the probability of next hop neighbour going out from or 2nd hop neighbour coming in direct communication range of *H*.

### 3.3. Optimum Probability for Route Update

The route update is generally not required by the static networks. However, for the networks with high mobility, lack of route update mechanism leads to significant decrease in efficiency. Hence, we can state that need of route update or probability of change in link status is proportional to the value of change in topology (layout, number of nodes in direct range, etc.). Thus, the change in topology is directly proportional to *P*[*N*_1_(out)  or  *N*_2_(in)].

As a test case, we can consider the example of routing strategy of minimum hop count towards the destination. This approach will have higher value of *P*(2) as each node will try to find the next hop neighbour closest to its communication boundary. The same approach being adapted by the next hop neighbour will tend to select 2nd hop neighbour, also at farthest distance. This intention will cause lower value of *P*(3) for 2nd hop neighbour. On the other hand, the behaviour of a routing strategy based on maximum stable link will have lower *P*(2) and higher *P*(3) value. However, this behaviour may not stand true for strategies which do not depend on node distance, such as minimum cost or load balance.

**Figure 3:**
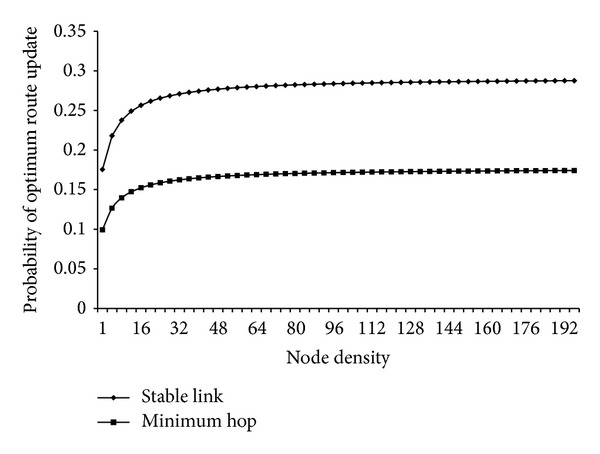
Optimum link probability.

Equation (4) provides a very interesting result as shown in Figure 3. The *x*-axis shows the node density, whereas *y*-axis shows the probability of optimum route update due to link breakage with next hop neighbour or link establishment with 2nd hop neighbour. For the computation of combined probability behaviour curve of minimum hop count approach, pdf curve for linear distribution is used. Similarly, for maximum stable link approach, pdf curve for Centralized Pareto Distribution is used.

Both the curves drawn for routing approach of stable link and minimum hop count follow the same pattern and are monotonically increasing with increase in number of nodes *n*. It suggests that the requirement for route update is directly proportional to change in network topology.

The proposed model as defined in (4) is based on different communicating regions defined by (1). The time *t* is directly proportional to region size, as shown in (1), which is again directly proportional to number of nodes *n*, as shown in (4). Hence, we can draw two important conclusions, as:

- by keeping the *n* constant, the probability of change in network conditions for optimum route update increases with increase in the update interval *t*,
- by keeping the update interval *t* constant, the probability of change in network conditions for optimum route update increases with increase in *n*.

The above stated conclusions are the baseline factors for our adaptive route update strategy. The modification of current routing protocols by introducing adaptive route update can significantly improve the efficiency of the network. Hence, irrespective of route finding approach, the requirement for route update increases with increase in node density.

The reader may argue that with a decreasing node density, the existing route may not be the best route any more. However, it must not be ignored that if the node density is decreasing, then in an average sense, the probability of availability of a better route also decreases (as there are lesser choices). Subsequently, the same conclusion can also be stated as follows.

A higher node density requires a higher rate of change in the network conditions for a state where route update becomes an optimum choice, that is, where either next hop node is about to face link breakage or 2nd hop node has already come in next hop range.

## 4. Evaluation of Adaptive Routing Strategy

As described earlier, many other studies along with already stated results confirm that current predefined and fixed route update schemes may not perform efficiently under all circumstances. Resultantly, emphasis should be given to two basic but important issues related to routing strategies. A generic and flexible but realistic solution to these issues can enhance the routing efficiency. These issues include the following:

- which type(s) of metrics can be shared for route finding?
- How these metrics can be shared, that is, adaptively or through fixed scheme?

Literature review of routing algorithms reveals that major emphasis is given to first issue and improved solutions are still being suggested, whereas the other issue need reemphasis according to requirements of upcoming complex networks.

To utilize the results achieved through (4), we propose the term *adaptation* for route update strategy. Although adaptation has already been proposed for routing, optimization for adaptation has been generally neglected in our current static routing approaches. The principal design dissimilarity between adaptive and optimized adaptive approach is the use of different metrics according to runtime network conditions to adaptively find and update the best possible route. The optimized adaptation will use the *logical conditions* to find and update the route by abolishing the taxonomy of *reactive* and *proactive*. *Optimized adaptation* leads to use of metrics at runtime for route update strategy. Accordingly, metrics need to be redefined as per their use for route finding and update and maintenance, as a single metric may not perform optimally for all routing roles. In the light of (4), different threshold values for each metric or combination of metrics, computed on runtime, can develop adaptive route update approach.

As already described, a localized metric can show more promising results as compared to end-to-end metric for local route repair. Analysis of networks with large topology changes emphasizes use of different types of metrics for efficient routing strategy, for example, QoS related metrics, position related metrics, and PHY layer metrics. These routing metrics, which are also cross layer in design, independently define changes in network topology. The different threshold values for each metric or combination of metrics, computed on runtime, can be used for adaptive route update. However, routing strategies with multimetrics schemes can also be researched for the purpose.

QoS related metrics such as throughput, delay, and packet pair delay are often used in routing schemes [11]. Considering the use of local route repair, QoS metrics for next hop only performs more efficiently than end-to-end metrics.

The use of position related metrics [8], such as neighbouring nodes, average node distance, and number of neighbours, has significant importance for networks such as VANETs. These metrics are also considered for geographical addressing. For highly fluent networks, one hop neighbours list provides more realistic results for change in network topology than average neighbour count or neighbour distance.

The use of physical layer metrics, such as SINR and received power, require more complicated algorithm designs but provide more stable routes [19]. Being a combination of received signal strength, noise, and interference, SINR provides more promising results than considering received signal strength only.

To verify our research for different possible scenarios of VANETs, we performed various simulations in NS-2. After comprehensive verification of the proposed scheme, we tested it against some state-of-the-art routing protocols.

As a test platform for the adaptive route update approach, we modified standard AODV routing protocol and named it as Adaptive AODV (AAODV). AODV, being a reactive routing protocol, update its route on link breakage only. However, it continuously shares HELLO messages to learn about its neighbours. AODV was modified in a manner, where each node was defined to continuously measure the metric for route update, for example, SINR, and adaptively update its route if change in metric value achieves a predefined threshold.

As a test metric, one hop neighbours list, next hop throughput, and SINR were selected. All three metrics showed significant improvement in overall performance. Selection of SINR can be argued considering real life implementation, as one hop neighbours list can easily be computed using standard HELLO messages. However, with the advancement in cross layer design, the use of physical layer metrics is emerging. Hence, considering the slight edge in performance, SINR was selected for subsequent evaluation.

SINR also indirectly shows the change in node topology around *H*. The change in received signal power can show the change in either signal (*S*) strength or noise (*N*) plus interference (*I*) strength. Increase in SINR means either reduction in interfering node density or next hop node distance and vice versa. Each node can compute SINR value around itself for each next hop neighbour, using formula [13]:

(5) $$\begin{array}{c} \text{SINR}=\frac{S}{N+I}=\frac{P_{t}h_{t}l\left(r\right)}{N+\sum_{\iota \epsilon \phi_{s}}^{}\left(P_{i}h_{i}lK\left|x_{i}^{-a}\right|\right)}, \end{array}$$

where *P*_*t*_ = Transmitted power (constant), *h*_*t*_ = Channel gain of transmitter (constant), *P*_*i*_ = Transmitted power from *i*_th_ node (constant), *r* = Transmitter to receiver distance, and *x*_*i*_ = Distance between *i*_th_ interfering node and receiver.

For the simulation purpose, highly mobile nodes (20–250) moving at variable velocities (1–130 kmph) were deployed in sparse area. Nodes were converged from highways to a single road crossing. Node density was gradually increased by converging all nodes within one hop region. After reaching the road crossing region, all nodes were forced to adapt a temporary static behaviour. After staying for a while in one hop region, all nodes moved in different directions.

For the comparative study of different routing protocols against optimized AAODV, 28 different simulations were run for 10 repetitions each. 7 different topologies were simulated under different scenarios and conditions by varying the number of nodes. Nodes were randomly selected with random timings to generate TCP and UDP traffic.

Using the percentage change in various metrics values, the optimum local route update conditions can be worked out. To find the optimum threshold value for route update, quality of service (QoS) parameters were measured by keeping different threshold values of the metric, under different node densities. After acquiring the coarse grained optimum threshold values of SINR, we compared our adaptive approach with other state-of-the-art routing protocols. The parameters considered for the simulations were as under:

- number of active nodes: 4, 8, 16, 32, 56, 64, and 128,
- number of passive nodes: 9 and 18,
- MAC protocol: IEEE 802.11p,
- number of lanes: 2, 4, and 8,
- node speeds: 16, 32, 50, 65, 80, 96, 112, and 130 kmph,
- comparative protocols: AAODV, AODVv2 (DYMO), OLSRv2, FROMR, and XORi.

SINR value can vary from zero to approaching infinity (very high), that is, either signal strength approaches zero (near link breakage) or approaches infinity (no noise and interference). Hence, different threshold values of SINR change were considered from 1 to 1 Billion. QoS parameters were measured by keeping different threshold values of SINR to find the optimum values under different node densities. Optimum threshold values for different node densities were computed at which adaptive route update provided best results.

**Figure 4:**
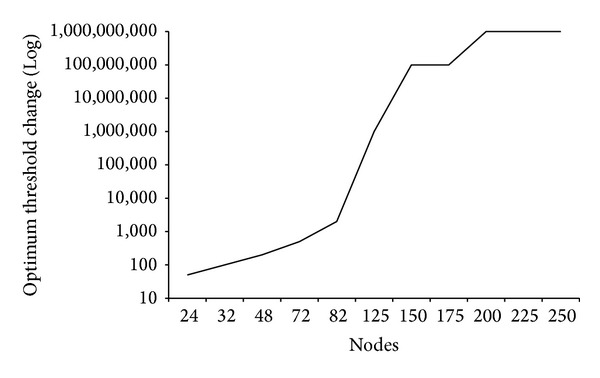
Optimum threshold change.

Figure 4 shows the graph of optimum change in SINR threshold for different node densities. The *x*-axis of the graph shows the combination of different node densities, whereas *y*-axis shows the percentage value of change at which local route update provided maximum data transfer for the particular topology. As the difference between lowest and highest threshold value is too large, the logarithmic scale was selected for better analyses of the curve. It can be observed that the value of optimum change threshold increases with increase in the node density. This result shows that the optimum threshold is dependent on total nodes involved in data transfer. Minimum value of change in SINR (50%) was observed for 24 nodes, whereas maximum value of 1 Billion was recorded for 250 nodes topology.

Interestingly, the optimum threshold curve for the SINR change follows the same pattern as the mathematical model in (4). The little variations can be considered due to randomness in topologies and variations in routing approach. Similar to Figure 2, the difference in threshold values for sparse topologies is more as compared to the dense ones. This result confirms the analysis that optimum threshold value is dependent on active nodes within given area.

Optimum threshold value starts from lower values and then increases to achieve some maximum value. After attaining the higher threshold values for higher node densities, the change in optimum values decreases significantly and the curve becomes flat. The threshold curve flattens for large node densities. Although being very low, increasing trend continues for all values.

**Figure 5:**
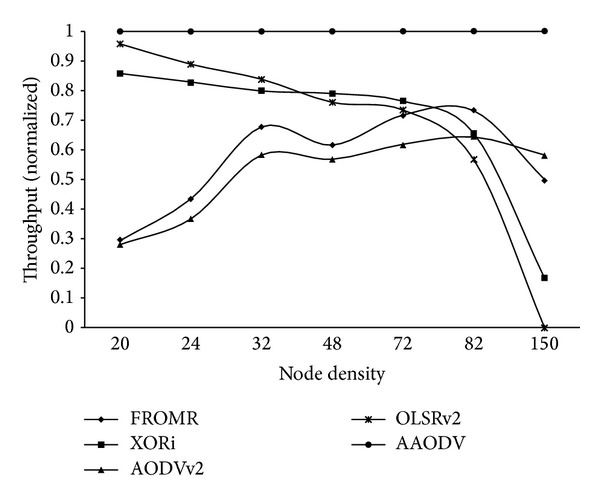
Throughput comparison of proposed approach (independently normalised for each topology).

**Figure 6:**
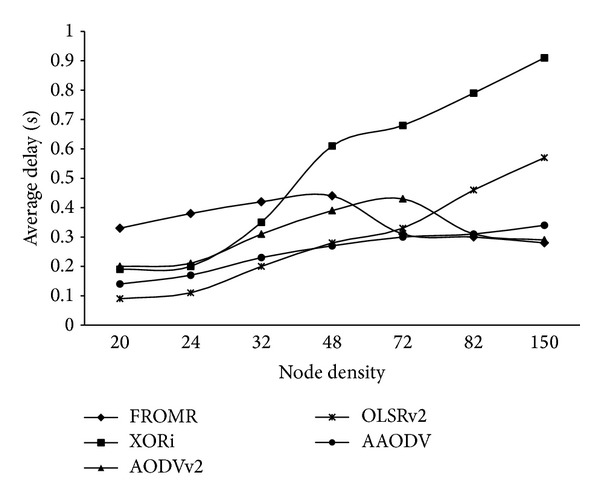
Delay comparison of proposed approach.

After attaining the coarse grained optimum threshold values of selected test metrics for route update, the comparison of optimized AAODV with other standard protocols was done. Figures 5 and 6 show the comparison of AAODV against AODVv2 (DYMO) OLSRv2, FROMR [26], and XORi [27]. AODVv2 and OLSRv2 are designed as routing protocols for Mobile Ad hoc Network (MANET), whereas the latter two are specifically designed for VANETs. In Figure 5, the *x*-axis of the graph shows the combination of different node densities. The *y*-axis shows the average of ratio of normalized throughput independently computed for each node density and topology, for different protocols against AAODV.

The comparison of curves for the low node densities shows better performance by XORi, OLSRv2, and AAODV. With change in topology, XORi and OLSRv2 timely updated their routes. Resultantly, due to low overheads at lower node density, both protocols showed comparative performance with AAODV. However, due to drastic increase in overheads at higher node densities, both protocols showed significant reduction in performance against AAODV.

On increasing the node density, higher contention and control overheads caused lack of sufficient bandwidth for control messages for OLSRv2. The curve confirmed the previous analysis of Figure 1.

From other two protocols, FROMR and AODVv2 performed equivalently to each other. However FROMR had a slight edge due to use of alternate available routes. For low node densities, both routing protocols remained inefficient due to nonadaptation to topology changes. By increasing the node density, more options were made available for route to destination. However, performance of both protocols remained low even for higher node densities. For all node densities, both protocols did not optimize their routing tables on availability of more suitable path. Resultantly, AAODV outperformed in all cases.

On increasing the node density further, the gap between curves of both protocols and AAODV started decreasing. Due to design limitation of three retries, higher contention rate caused repeated false route failures for these protocols. Forced route error messages caused route update, even in presence of old route. Repeated unintentional route update improved the performance of reactive protocols. The same behaviour can be seen at node density of 150 active nodes. However, the unintentional update did not cause all nodes to update their route. Similarly, the behaviour cannot be guaranteed under all topologies.

Figure 6 shows the comparison of delay for selected protocols. The *x*-axis of the graph shows the combination of different node densities. The *y*-axis shows the average of end-to-end delay for each topology. We can observe that for the low node densities, OLSRv2 offered minimum delay owing to its simple proactive design. However on increasing the node density, increased overheads caused significant increase in end-to-end delay. For higher node densities, AODVv2 showed minimum delay due to its proactive design and false route update, as already defined.

Use of adaptive route update can make any routing protocol more efficient. Optimization of adaptive route update can be performed on different node densities and type of networks. However, the threshold level for optimized adaptive route update may vary for different networks.

## 5. Conclusion

Routing protocols can be divided into many categories according to the algorithm and modifications proposed against other protocols. Using different metrics, routing protocols can be grouped into three categories according to routing metric sharing method. These types include periodic topology sharing, event based topology sharing, and their derivatives (hybrid and history oriented).

Behaviour of any routing algorithms differs for static and mobile network scenarios. Contrary to static networks, mobile wireless networks suffer from sudden link breakages due to topological changes, change in node densities, and reduction in average link capacities. Resultantly, any single protocol may not perform well under all scenarios and conditions. The analysis of routing strategies proves their inefficiency for complex networks.

Without incorporating adaptive route update, existing protocols can provide satisfactory results for networks with limited topology changes and limited number of nodes. However, for large scale networks or networks involving rapid topology changes, current routing strategies will face performance issues.

We developed a mathematical model to describe the behaviour of changes in network conditions. The curves of the model provide a very interesting result as the probability for optimum route update is directly proportional to time interval and node density. Rapid topology changes demand adaptive use of runtime intelligence for route update. The proposed adaptive route update scheme which can be implemented with any baseline routing algorithm will allow nodes to locally optimize their routes. Hence, adaptive use of runtime conditions will replace the terms reactive or proactive protocols with logical conditions to find the most optimum route at any given time.

Regardless of baseline routing approach, adaptive route update, based on different metrics, can make a routing protocol more efficient than a routing protocol with static route update approach. As a future work, we intend to extend theoretical analysis of the proposed approach, including complexity analysis. We also intend to modify some other routing protocols according to the proposed approach, to verify the improvements against their standard versions.

## Figures and Tables

**Table 1:** Routing update strategy comparison.

Primary factor	Options	Routing preference
Application needs	Control messages	Reactive
	Broadcast	Reactive

Node deployment	Ad hoc	Proactive
	Hybrid (Clustered biased)	Hybrid
	Hybrid (Infrastructure biased)	Reactive

Mobility model	Random	Proactive
	Bidirectional probabilistic	Reactive
	Multidirectional probabilistic	Hybrid

Node density	High	Reactive
	Low	Proactive

Scalability	High	Reactive
	Low	Proactive

Relative mobility	High	Hybrid
	Medium	Proactive
	Low	Reactive

QoS requirement	Throughput	Reactive
	Jitter	Proactive
	Delay	Proactive

**Table 2:** Possibility of link breakage in *R*2.

Host node status	Next hop neighbour status	Neighbour node movement direction	Link breakage possibility	Probability of going out
Moving	Moving	Same	No	1/6
Moving	Moving	Opposite	No	
Moving	Static		No	
Static	Moving	Same	Yes	
Static	Moving	Opposite	No	
Static	Static		No	

## References

[1] Rasheed A, Qayyum A, Ajmal S Security architecture parameters in VANETs.

[2] Xeros A, Lestas M, Andreou M, Pitsillides A Adaptive probabilistic flooding for Information Hovering in VANETs.

[3] Sun MT, Feng WC, Lai TH, Yamada K, Okada H, Fujimura K GPS-based message broadcast for adaptive inter-vehicle communications.

[4] Tseng YC, Ni SY, Chen YS, Sheu JP (2002). The broadcast storm problem in a mobile ad hoc network. *Wireless Networks*.

[5] Dahiya A, Chauhan R (2010). A comparative study of manet and VANET environment. *Journal of Computing*.

[6] Mohseni S, Hassan R, Patel A, Razali R Comparative review study of reactive and proactive routing protocols in MANETs.

[7] Shinohara Y, Chiba Y, Shimonishi H An adaptive multipath routing algorithm for maximizing flow throughputs.

[8] Li F, Wang Y (2007). Routing in vehicular ad hoc networks: a survey. *IEEE Vehicular Technology Magazine*.

[9] Slavik M, Mahgoub I, Rathod M Statistical broadcast protocol design with WiBDAT: wireless Broadcast design and analysis tool.

[10] Shah RC, Wolisz A, Rabaey JM On the performance of geographical routing in the presence of localization errors.

[11] Rainer Baumann MS, Heimlicher S, Weibel A A survey on routing metrics.

[12] Karp B, Kung HT GPSR: Greedy Perimeter Stateless Routing for wireless networks.

[13] Goldsmith A (2005). *Wireless Communications*.

[14] Mohammad SA, Rasheed A, Qayyum A (2011). VANET architectures and protocol stacks: a survey. *Communication Technologies for Vehicles*.

[15] Zarei B, Zeynali M, Majid Nezhad V Novel cluster based routing protocol in wireless sensor networks. *International Journal of Computer Science Issues*.

[16] Zhao J, Cao G VADD: Vehicle-assisted data delivery in vehicular ad hoc networks.

[17] Zhang M, Wolff RS Border node based routing protocol for VANETs in sparse and rural areas.

[18] Leontiadis I, Mascolo C GeOpps: Geographical opportunistic routing for vehicular networks.

[19] Rasheed A, Ajmal S 3D-a doppler, directivity and distance based architecture for selecting stable routing links in VANETs.

[20] Slavik M, Mahgoub I (2013). Spatial distribution and channel quality adaptive protocol for multi-hop wireless broadcast routing in VANET. *IEEE Transactions on Mobile Computing*.

[21] Kim Y, Lee JJ, Helmy A (2004). Modeling and analyzing the impact of location inconsistencies on geographic routing in wireless networks. *ACM SIGMOBILE Mobile Computing and Communications Review*.

[22] Rasheed A, Zia H, Hashmi F, Hadi U, Naim W, Ajmal S (2013). Fleet & convoy management using VANET. *Journal of Computer Networks*.

[23] Shafiee K, Leung VCM (2011). Connectivity-aware minimum-delay geographic routing with vehicle tracking in VANETs. *Ad Hoc Networks*.

[24] Al-Rabayah M, Malaney R (2012). A new scalable hybrid routing protocol for VANETs. *IEEE Transactions on Vehicular Technology*.

[25] Barr R, Haas ZJ, Van Renesse R (2005). JiST: an efficient approach to simulation using virtual machines. *Software*.

[26] Wu CS, Hu SC, Hsu CS Design of fast restoration multipath routing in VANETs.

[27] Oliveira R, Garrido A, Pasquini R Towards the use of XOR-based routing protocols in vehicular ad hoc networks.

[28] Saleet H, Langar R, Basir O, Boutaba R Adaptive message routing with QoS support in vehicular Ad Hoc networks.

[29] Zeadally S, Hunt R, Chen YS, Irwin A, Hassan A (2012). Vehicular ad hoc networks (VANETs): status, results, and challenges. *Telecommunication Systems*.

[30] Jaap MBS, Wolf L Evaluation of routing protocols for vehicular ad hoc networks in city traffic scenarios.

[31] Song T, Xia W, Song T, Shen L A cluster-based directional routing protocol in VANET.

[32] Murray D, Dixon M, Koziniec T An experimental comparison of routing protocols in multi hop ad hoc networks.

[33] Namboodiri V, Gao L (2007). Prediction-based routing for vehicular Ad Hoc networks. *IEEE Transactions on Vehicular Technology*.

[34] Clausen T, Dearlove C, Jacquet P The optimized link state routing protocol version 2.

